# Metabolomics to Decipher the Chemical Defense of Cereals against *Fusarium graminearum* and Deoxynivalenol Accumulation

**DOI:** 10.3390/ijms161024839

**Published:** 2015-10-20

**Authors:** Léa Gauthier, Vessela Atanasova-Penichon, Sylvain Chéreau, Florence Richard-Forget

**Affiliations:** 1 Euralis, Domaine de Sandreau, 6 chemin de Panedeautes, Mondonville CS 60224, 31705 Blagnac Cedex, France; E-Mail: lea.gauthier@bordeaux.inra.fr; 2 INRA, UR1264 MycSA, 71 avenue Edouard Bourleaux, CS20032, 33882 Villenave d’Ornon Cedex, France; E-Mails: vatanaso@bordeaux.inra.fr (V.A.-P.); sylvain.chereau@bordeaux.inra.fr (S.C.)

**Keywords:** toxigenic fungi, plant resistance, metabolites, phenylpropanoids, terpenoids, fatty acids derivatives

## Abstract

*Fusarium graminearum* is the causal agent of *Fusarium* head blight (FHB) and Gibberella ear rot (GER), two devastating diseases of wheat, barley, and maize. Furthermore, *F. graminearum* species can produce type B trichothecene mycotoxins that accumulate in grains. Use of FHB and GER resistant cultivars is one of the most promising strategies to reduce damage induced by *F. graminearum*. Combined with genetic approaches, metabolomic ones can provide powerful opportunities for plant breeding through the identification of resistant biomarker metabolites which have the advantage of integrating the genetic background and the influence of the environment. In the past decade, several metabolomics attempts have been made to decipher the chemical defense that cereals employ to counteract *F. graminearum*. By covering the major classes of metabolites that have been highlighted and addressing their potential role, this review demonstrates the complex and integrated network of events that cereals can orchestrate to resist to *F. graminearum*.

## 1. Introduction

*Fusarium* head blight (FHB) of wheat and barley and Giberella ear rot (GER) of maize are two devastating fungal diseases affecting cereal crops worldwide [1]. FHB and GER are both caused by the same *Fusarium* species with *F. graminearum*, *F. culmorum*, *F. avenaceum* and *F. poae* being the most predominant in Europe. FHB and GER lead to huge economic losses, resulting from reduced yields, reduced grain quality and contamination of grains with mycotoxins.

Type B trichothecenes (TCTB) are the most frequently encountered *Fusarium* mycotoxins in Europe [2]. TCTB mycotoxins include deoxynivalenol (DON) and its acetylated forms 15-acetyl-4-deoxynivalenol and 3-acetyl-4-deoxynivalenol (15-ADON and 3-ADON) along with nivalenol (NIV) and its acetylated form fusarenone X (FX). TCTB mycotoxins have various acute and chronic effects on humans and animals. TCTB mycotoxins were shown as the primary cause of alimentary toxic aleukia, which has been responsible for the death of thousands people in URSS in the 1940s [3]. Currently, the most worrying concern with TCTB mycotoxins does not result from short term exposure to high concentrations but more to a prolonged daily exposure leading to chronic health effects. Consequently, maxima for DON levels in foodstuffs have been set up in Europe since June 2005 (Commission regulation EC number 856/2005 amended in July 2007/EC number 1126/2007). TCTB mycotoxins are highly heat stable molecules and cannot efficiently be destroyed by current food processes [4]. As a result, the better way to limit TCTB mycotoxins incidence in food and feed is to minimize their production in crops before harvest.

In order to reduce the risks of accumulating TCTB mycotoxins in kernels, several cultural control practices have been shown to be efficient. These control strategies rely on reducing levels of primary pathogen inoculum through management strategies such as crop rotation, tillage, and use of chemicals and also on breaking the fungal disease cycle by adapting the sowing period or using resistant hosts [5]. More recently, integrated management studies have demonstrated the improvements that can be gained by combining multiple control strategies [6]. Plant breeding strategies are among the most promising and performing approaches to fight against the mycotoxin problem in the short to long-term range and certainly one of the most important pillars of these integrated disease management programs [7].

Plant resistance to FHB is a highly complex quantitative trait controlled by multiple genes, depending on environmental and genotype x environment interactions [8]. FHB resistance was first broken down into two components: type I resistance that operates against initial infection, and type II resistance that operates against the spread of the pathogen within the host [9]. Later, three additional components were distinguished: resistance to kernel infection (type III), tolerance to infection (type IV), and resistance to DON accumulation (type V) [10,11]. Over 100 Quantitative Trait Loci (QTL) for FHB resistance in wheat have been identified. Buerstmayr *et al.* [12] reviewed the stable QTL for FHB resistance found by previous research. QTL for FHB disease were found on all wheat chromosomes except chromosome 7D and the most repeatable ones are located on chromosomes 3BS, 5AS and 6BS. Compared to wheat, there are very few FHB resistance sources in barley and less than 30 QTL, distributed over all seven barley chromosomes, have been identified [8]. Several of these QTL are linked with heading date, plant height and spikelet morphology such as kernel discoloration [13,14,15]. Regarding GER resistance and DON accumulation in maize, a few genetic studies have been conducted so far. One of the first QTL study was published in 2011 by Martin *et al.* [16] who identified six QTL for GER resistance and four QTL for low DON accumulation. Despite such significant progress in the understanding of the genetic bases of resistance to *Fusarium* (particularly for wheat), knowledge remains partial, and selection for FHB and GER resistance is still challenging. The polygenic control of FHB and GER resistances that involve a complex and interacting network of signaling pathways is certainly the major obstacle to successful selection [8]. In combination with genetic approaches, biochemical ones can provide valuable insights in the mechanisms crops employ against *F. graminearum* and its production of mycotoxins. Several mechanisms have been actually associated with the ability of crops to detoxify DON. These detoxification processes involve enzymatic chemical modifications catalyzed by a set of UDP-glycosyltransferases, gluthatione-*S*-transferases or cytochrome P450 mono-oxygenases [17]. The recent findings of Kluger *et al.* [18] demonstrate the diversity of DON detoxification products that can occur in resistant wheat lines and strongly corroborate the correlation of DON detoxification pathways with a major QTL for FHB resistance, *Fhb1*. Otherwise, a large set of metabolites, pre-formed, constitutive as well as inducible defense metabolites could play a pivotal role in the resistance of cereals against pathogenic fungi. According to Balmer *et al.* [19], these metabolites can be roughly divided in three major groups: alkaloids, isoprenoids and shikimates. There have been many attempts to identify the key metabolites involved in resistance to FHB or GER and low DON accumulation. Mainly based on a comparative approach of the metabolite compositions of resistant and susceptible cultivars, challenged or not with *F. graminearum*, these attempts have implemented targeted analytical tools and more recently metabolomic developments [20,21,22,23,24,25,26,27,28,29,30,31,32].

Metabolomics is reported as a comprehensive, nonbiased, high throughput analysis of complex metabolite mixtures ideally allowing the identification and quantification of every individual metabolite [33]. Most studies are based on gas chromatography or Ultra High Performance Liquid Chromatography coupled to Mass Spectrometry (GC-MS or UPLC-MS) [34,35,36,37]. However, although these techniques using a mass analyzer provide good selectivity and sensitivity, they usually cannot differentiate between isomeric configurations and are highly sensitive to matrix effects [34]. These constraints can be partially overcome with the use of Nuclear Magnetic Resistance (NMR) in addition to MS-based approaches [38,39,40].

The aim of this review is to provide recent insights on the key plant metabolites that can be involved in the resistance of cereals to *Fusarium* and the experimental evidences of their ability to restrain *Fusarium* growth and DON production.

## 2. A Large and Diverse Set of Metabolites Potentially Involved in Biochemical Resistance to FHB Spread Have Been Pinpointed through the Achievements of Metabolomic Approaches

**Figure 1:**
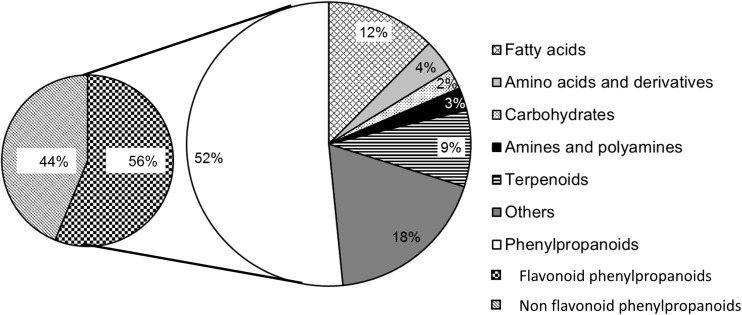
Chemical diversity of metabolites that have been pinpointed for their potential contribution to FHB resistance through the achievement of metabolomic studies [20,21,22,23,24,25,26,27,28,29,30,31,32] and have been putatively identified.

Up to date, the metabolomic approaches that have been applied to phenotype resistance against *F. graminearum* and *F. culmorum* have been mainly restricted to wheat and barley, using genotypes with contrasted levels of resistance, classified as resistant or susceptible, and comparing mock-inoculated *versus* pathogen-inoculated plants. In addition to *F. graminearum* inoculation, Paranidharan *et al.* [32] and Warth *et al.* [41] also used DON injection into the middle florets of spikelets to decipher the mechanism of plant resistance to the toxin. The host response to DON was also addressed by Kumaraswamy *et al.* [29] and Gunnaiah and Kushalappa [25] who used inoculations with a DON-producing isolate and a DON-non producing *F. graminearum* isolate with loss of function of *Tri5* gene. All these approaches of metabolomics are summarized in Table 1 together with the experimental designs and methodologies used by the authors. They have led to the characterization of a large set of metabolites with concentrations that were significantly higher in the resistant genotypes than in the susceptible ones. In most of the publications gathered in Table 1, these metabolites were referred to resistance-related (RR) metabolites. In some studies, these RR metabolites have been classified into two groups: RR compounds resulting from mock inoculations were classified as constitutive (RRC) whereas metabolites that increase in concentration after pathogen inoculation were called RR induced metabolite (RRI) [24,26,28]. However, the concept of “resistant-related” metabolites must be considered with caution since, in most of the studies gathered in Table 1, the experimental design was based on the comparison of a set of unrelated germplasms, which is not sufficient to provide the basis for such a claim. In reality, the differences reported in the metabolic profiles of the considered genotypes may actually be confounding with cultivar background effects. The use of near isogenic lines, as done in the study of Guannaiah *et al.* [26], represents the most suitable strategy to reach conclusive evidences. Moreover, since environment, cultivation practices, developmental stage and the chemotype of the inoculated *F. graminearum* strain [29] are additional factors with significant impact on the metabolic profiles of kernels and their response to the pathogen, the data delivered in each of the metabolomic studies reported in Table 1 should not be dissociated from the experimental designs that led to their discovery. Lastly, it should be borne in mind that chemical identification remains a significant bottleneck in plant metabolomic studies and that most of the peaks detected using mass spectrometry cannot be assigned to identified metabolites. In most of the studies gathered in Table 1, metabolites were putatively identified by comparison of spectra with reference spectra contained in several metabolite databases including METLIN, NIST, GMD [20,30,32]. Criteria for metabolite assignment included (i) accurate mass match with database; (ii) fragmentation pattern match with databases and (iii) determination of the number of carbons in the molecular formulae based on isotope ratio. In few studies, metabolite assignments were confirmed by spiking the samples with standard of the suspected compound [22,29,31]. As shown on Table 1, the number of metabolites with a putative identification significantly varies according to the experimental design and the applied analytical strategy, ranging from 10 in the HNMR study of Browne and Brindle [22] to more than 150 in the study of Kumaraswany *et al.* [30] based on LC-ESI-LTQ Orbitrap analysis. As indicated in Table 1 and Figure 1, the metabolites highlighted for their potential contribution to resistance to FHB spread can be roughly categorized in seven chemical groups, according to their putative chemical structure. These seven chemical groups can be ranked according to the number of metabolites identified in each group as follows: flavonoid phenylpropanoids, non-flavonoid phenylpropanoids, fatty acids, terpenoids, amino acids and derivatives, amines and polyamines and carbohydrates. An additional group designated as “others” in Figure 1 gathers metabolites that have been putatively assigned to different chemical groups including organic acids, xanthonoids and benzoxazinoids. Surprisingly, only one benzoxazinoid derivative, the 6-methoxybenzoxazolin-2[3*H*]-one, was putatively identified in the thirteen studies used for this overview, while these cereal metabolites have been extensively studied for their antimicrobial efficiency and their key role in defense of maize against various pathogens [42,43]. Most of the putatively identified metabolites derive from the shikimate, acetate-mevalonate and methylerythritol phosphate pathways, which are the three main plant metabolic pathways involved in cereal defense against pathogens and pests [19]. According to Gunnaiah and Kushalappa [25], the chemical defense against fungal pathogens including DON producing *Fusarium* species is linked to three main mechanisms of resistance: cell wall reinforcement through the deposition of lignin, production of antimicrobial compounds and specific induction of defense signaling pathways.

**Table 1:** Overview of metabolomic studies addressing the mechanisms of *Fusarium* head blight (FHB) resistance in wheat and barley.

Pathosystem		Part of the Plant; Stage of Inoculation ^ab^; Harvesting ^a^	Technology	Database	Putatively Identified Metabolites Linked to FHB Resistance	Main Chemical Groups	Reference
Plant	Pathogen						
Wheat. 2 cultivars: 1 susceptible (Roblin); 1 resistant (Sumai3)	*F.* *graminearum*	Spikelets; Inoculation GS = 60–69 (anthesis); Harvested at 24 hai	GC-MS	NIST	55	Carbohydrates; Fatty acids; Phenylpropanoids	[27]
Wheat. 121 genotypes with different levels of FHB resistance	none	Leaf and stem; no inoculation Harvested at 14 days of growing	^1^H NMR	Identification by spiking with standards	10	Amines; Amino acids; Carbohydrates	[22]
Wheat. 6 cultivars with different levels of FHB resistance	*F.* *graminearum*	Spikelets; Inoculation GS = 60–69 (anthesis); Harvested at 24 hai	GC-MS	GMD; NIST	45	Amino acids; Carbohydrates; Fatty acids; Organic acids; Phenylpropanoids; Polyamines	[28]
Wheat. 2 cultivars: 1 susceptible (Roblin); 1 resistant (Sumai3)	*F.* *graminearum* or DON	Spikelets; Inoculation GS = 65 (anthesis); Harvested at 48 hai	GC-MS	GMD; NIST	47	Amino acids; Carbohydrates; Fatty acids; Organic acids; Phnelypropanoids; Polyamines	[32]
Barley. 2 cultivars: 1 susceptible (Stander); 1 resistant (Chevron)	*F.* *graminearum*	Spikelets; Inoculation GS = 65–73; Harvested at 48 hai	LC-ESI-LTQ- Orbitrap	METLIN; PubChem; KNApSAcK; HMDB; MoTo	47	Amino acids; Fatty acids; Phenylpropanoids	[20]
Barley. 6 genotypes: 5 resistant (Chevron + 4 others); 1 susceptible (Stander)	*F.* *graminearum*	Spikelets; Inoculation GS = 71–75; Harvested at 72 hai	LC-ESI-LTQ-Orbitrap	Not cited	130	Fatty acids; Phenylpropanoids; Terpenoids	[21]
Barley. 2 genotypes: 1 susceptible; 1 resistant	*F.* *graminearum*	Spikelets; Inoculation at GS = 65–73 (anthesis to early milk stage); NA	LC-ESI-LTQ-Orbitrap	METLIN; KNApSAcK; MassBank; McGill-MD; KEGG; HMDB	53	Fatty acids; Phenylpropanoids	[30]
Barley. 6 genotypes: 1 susceptible; 5 resistant	*F. graminearum*	Spikelets; Inoculation at GS = 65 (anthesis to early milk stage); Harvested at 72 hai	LC-ESI-LTQ-Orbitrap	Databases not cited Identification by spiking with standards	38	Fatty acids; Phenylpropanoids	[31]
Wheat. 2 near Isogenic Lines with susceptible and resistant alleles of *Fhb1*	*F.* *graminearum*	Rachis and spikelets; Inoculated at anthesis; Harvested at 72 hai	LC-ESI-LTQ-Orbitrap	METLIN; KNApSAcK; PMN; LIPIDMAPS; KEGG; McGill-MD	Rachises: 87 Spikelets: 57	Fatty acids; Phenylpropanoids; Terpenoids	[26]
Black barley. 2 lines: 1 susceptible; 1 resistant	*F.* *graminearum*	Spikelets; Inoculation at GS = 65–73 (anthesis to early milk stage); Harvested at 72 hai	LC-ESI-LTQ-Orbitrap	Not cited	74	Amino acids; Carbohydrates; Fatty acids; Phenylpropanoids	[29]
Yellow barley. 2 lines: 1 susceptible; 1 resistant				Identification by spiking with standards			
Barley. 5 lines: 1 susceptible; 4 resistant	*F. graminearum*	Spikelets; Inoculation at 50% anthesis; Harvested at 72 hai	LC-ESI-LTQ-Orbitrap	PlanyCyc; METLIN; KNApSAcK; KEGG	76	Alkaloids; Fatty acids; Hydroxycinnamic acids; Phenylpropanoids; Terpenoids	[24]
Barley. 9 varieties: Aktiv; Gladys; Henrike; Lilly; Radegast; Sebastian; Signora; Sladar; Tolar	*F.* *culmorum*	NA; Inoculated at the beginning of heading Harvested at 11 dai	UHPLC-Q-TOF-MS	PlanyCyc; METLIN; MassBank	13	Fatty acids; Phenylpropanoids	[23]
Wheat 2 cultivars: 1 susceptible (Roblin); 1 resistant (Sumai3)	*F.* *graminearum*	Rachis Inoculated GS = 65 (anthesis) Harvested at 3 dai	LC-ESI-LTQ-Orbitrap,	METLIN; MassBank; MS2T	133	Amino acids; Carbohydrates; Phenylpropanoids; Terpenoids	[25]

^a^ hai: hours after inoculation, dai: days after inoculation, NA: not available; ^b^ GS: Growth Stage on the Zadocks scale [44].

## 3. Chemical Groups Potentially Involved in Chemical Defense against DON Producing *Fusarium* Species

### 3.1. Metabolites Derived from the Phenylpropanoid Pathway

#### 3.1.1. Flavonoid Phenylpropanoids

As illustrated on Figure 1, metabolites putatively associated with the phenylpropanoid pathway are frequently reported for their potential contribution to chemical defense against *F. graminearum* and the production of DON. More than 340 different compounds have been highlighted through the achievement of the metabolomic studies gathered in Table 1 and 178 were putatively assigned to phenylpropanoid compounds. Plant phenylpropanoids encompass several classes of structurally diverse compounds synthesized from the amino acid phenylalanine, including flavonoids such as anthocyanins, flavones, flavonols, flavanones, flavanols, isoflavones, isoflavanones, isoflavonols and chalcones, and nonflavonoids such as phenolic acids, phenolic alcohols, phenolic aldehydes, stilbenes, lignans and coumarins (Table 2 and Table 3). Among the 178 metabolites putatively assigned to phenylpropanoids, more than 56% were supposed to belong to the flavonoid class (Figure 1). Numerous data support the involvement of phenylpropanoid compounds in plant resistance to fungal pathogens [45], which mainly results from their antibiotic properties, their key role as plant defense mediators and their participation to cell wall lignification. Some phenylpropanoids are produced constitutively and function as preformed antibiotics in non-host resistance to pathogens; they are designed as phytoanticipins. Others, which biosynthesis is induced in response to the pathogen ingress, participate to active plant defense mechanisms and can be classified as phytoalexins [46].

**Table 2:** Metabolites pinpointed for their potential contribution to FHB resistance and putatively assigned to flavonoid phenylpropanoid compounds.

Subgroup	Putative Name of Identity	Reference
Anthocyanin	Cynanidin 3-*O*-glucoside	[21]
	Malvidin 3-*O*-glucoside	[25]
	Pelargonidin 3-*O*-rutinoside	[29]
	Pelargonidin 3-*O*-sophoroside	[29]
Chalcon	2′,4′-Dihydroxy-6′-methoxychalcon	[29]
	Chalconaringenin 2′-rhamnosyl-(1->4)-xyloside	[25]
	Chalconaringenin 2′-xyloside	[29]
Flavanol	7,4′-Dihydroxyflavan	[21,23]
	7-Hydroxy-5,4-dimethoxy-flavan	[21]
	Catechin 3-*O*-α-l-rhamnoside	[21]
	Catechin 5,7,3′-trimethyl ether	[20]
	Catechin 7-*O*-apiofuranoside	[21]
	Catechin	[29,30]
	Catechin-4-ol 3-*O*-β-d-galactopyranoside	[20]
	Catechol glucoside	[21]
	Epicatechin-3-*O*-(3-*O*-methylgallate)	[25]
	Epicatechin 5-*O*-β-d-glucopyranoside-3-benzoate	[21]
	Epigallocatechin	[25]
	Gallocatechin-4β-ol	[21]
Flavanone	5-Hydroxy-7,8-dimethoxyflavanone 5-rhamnoside	[26]
	5-Hydroxy-7,4′-dimethoxy-6,8-di-c-prenylflavanone 5-*O*-galactoside	[25]
	5-*O*-Methylleridol	[21]
	5′-Prenylhomoeriodictyol	[25]
	6-Prenylnaringenin	[21]
	Dalpanin	[24]
	Exiguaflavanone	[31]
	Kuwanon L	[31]
	Mucronulatol-(4->6) naringenin	[21]
	Nallaflavanone	[1]
	Naringenin 7,4′-dimethyl ether	[29]
	Naringenin 7-*O*-(2′′,6′′-di-*O*-α-rhamnopyranosyl)-β-glucopyranoside	[25]
	Naringenin	[29]
	Naringenin-7-*O*-glucoside	[29,30]
	Naringenin 5,7-dimethyl ether 4′-O-xylosyl-(1->4)-arabinoside	[21]
	Tetrahydroxy-6,8-di-C-prenylflavanone	[29]
	3,5,7′-Trihydroxy-4-methoxyflavone	[21,30]
	5,6-Dimethoxyflavone	[26]
	5,4′-Dihydroxy-3,6,7,8,2′-pentamethoxyflavone	[21,30]
	5,7,2′-Trihydroxy-8,6′-dimethoxyflavone	[21,30]
	5,7,3′,5′-Tetrahydroxy-8,4′-dimethoxyflavone	[31]
	5-Hydroxy-3,6,7,4′-tetramethoxyflavone	[21]
	6-Prenylapigenin	[21]
	Alpinumisoflavone	[30]
	Alpinumisoflavone dimethyl ether	[21]
	Apigenin	[21,23]
	Apigenin 7-*O*-rutinoside	[25]
	Apigenin 7-*O*-β-d-glucuronide	[21]
	Calophyllolide	[31]
	Isoorientin 4′-*O*-glucoside-2′′-*O*-(*E*)-caffeate	[25]
	Isoscoparin	[21]
	Isovitexin 2′′-*O*-(6′′′-feruloyl) glucoside	[25]
	Isovitexin-7-*O*-glucosyl-2′′-*O*-rhamnoside	[24]
	Isovitexin-7-*O*-xyloside	[24]
	Lupinisoflavone G	[25]
	Skullcapflavone I 2′-(4′-*E*-cinnamoylglucoside)	[21]
	Tangeretin	[21]
	Tetrahydroxy-6,8-di-C-prenylflavone	[29]
	Tricin 7-rutinoside	[21]
	Ulexone B	[25]
	Vitexin	[29]
	Vitexin 2′′-*O*-(*E*)-ferulate	[21]
Flavonol	3,7-Di-*O*-methylquercetin	[29]
	6-Hydroxykaempferol 7,4′-dimethyl ether 3-sulfate	[31]
	Dihydroquercetin	[29]
	Isoscoparin 7-*O*-glucoside	[21]
	Kaemferide 5-glucoside-7-glucuronide	[31]
	Kaempferide 3,7-diglucoside	[31]
	Kaempferide 3-glucoside-7-rhamnoside	[20,21]
	Kaempferol 3-(2′′,3′′-diacetyl-4′′-*p*-coumaroyl rhamnoside)	[31]
	Kaempferol 3-(2′′-(*Z*)-*p*-coumaroyl rhamnoside)	[20]
	Kaempferol 3-(6′′-caffeoyl glucoside)	[20]
	Kaempferol 3-apiosyl-(1->4)-rhamnoside-7-rhamnoside	[21]
	Kaempferol-3-gentiobioside-7-rhamnoside	[20]
	Kaempferol-3-glucoside-7-rhamnoside	[21]
	Kaempferol-3-isorhamnoside	[31]
	Kaempferol-3-*O*-glucoside 7-*O*-rhamnoside	[21]
	Kaempferol-3-rhamnoside-7-glucosyl-(1->2)-rhamnoside	[21]
	Kaempferol-3-rhamnoside-7-xylosyl-(1->2)-rhamnoside	[20]
	Kaempferol-3-sophoroside-7-rhamnoside	[20]
	Kaempferol-3-xyloside	[21]
	Kaempferol-4′-methyl ether 3-neohesperioside	[31]
	Kaempferol-7,4′-dirhamnoside	[21,31]
	Kaempferol-3-*O*-α-rhamnoside	[20,21,23,25]
	Kaempferol-3-*O*-α-rhamnosyl glucoside	[21]
	Quercetin 3,7-dimethyl ether	[21,26]
	Quercetin 3-(6′′-acetylglucoside)	[31]
	Quercetin 3-*O*-methyl-7-*O*-galactoside	[21]
	Quercetin 5,7,3′,4′-tetramethyl ether	[30]
	Quercetin 7,3′,4′-trimethyl ether	[25]
	Quercetin	[30]
	Quercetin pentamethyl ether	[21]
	Quercetol	[29,30]
	Rhamnetin 3-*O*-rhamninoside	[21]
Isoflavone	2-Hydroxyisoflavone naringenin	[26]
	7-Hydroxy-4′-methoxyisoflavone	[31]
	7-Prenyloxy-3′,4′-dimethoxyisoflavone	[31]
Isoflavanone	(+−)-5-Deoxykievitone	[21]
	Sappanone A trimethyl ether	[21]
Isoflavonol	Methylophiopogonone B	[25]

As indicated in Table 2, almost 100 putative flavonoids that could contribute to the chemical arsenal of wheat and barley against *F. graminearum* were identified through the achievement of metabolomic studies. The majority of these metabolites correspond to glucoside derivatives of kaempferol and quercetin that belong to the flavonol class. In addition, few compounds of the flavanol (catechin and its derivatives), flavanone (naringenin), flavone (apigenin and vitexin derivatives) and anthocyanin (pelargonidin) classes were highlighted. These metabolomic data corroborate recent published studies that have indicated a significant induction of the expression of several genes involved in the biosynthetic pathway of flavonoids [47] and/or an increase in flavonol and flavanone concentrations [48] following wheat inoculation by *F. graminearum.* The main role ascribed to flavonoid in plant defense mechanisms results from their antioxidant properties [49,50,51], that allow them to reduce the production of and quench reactive oxygen species (ROS), generated by both the pathogen and the plant during infection. In addition, flavonoids are thought to participate to the reinforcement of plant structures and act as a physical barrier against fungal infection [52]. This role was recently supported by the findings of Venturini *et al.* [53] that strongly suggest the involvement of flavonoids in resistance to *F. verticillioides* through their contribution to kernels’ hardening. Flavonoids can also protect plant cell wall integrity upon fungal infection by inhibiting the activity of several plant cell wall degrading enzymes secreted by fungal pathogens to penetrate plant tissues [54]. Lastly, flavonoids are well known for their ability to inhibit fungal spore development and to restrain mycelium hyphae elongation. These antifungal activities were recently reviewed by Mierziak *et al.* [55] and according to Treutter [54], they directly result from the ability of flavonoids to irreversibly combine with nucleophilic amino acid in fungal proteins. Among the putative flavonoid compounds gathered in Table 2, naringenin, which was found to be much more abundant in some resistant wheat and barley cultivars than in susceptible ones [21,26,30], and has been reported as an efficient inhibitor of *in vitro* growth of *F. graminearum* [20], could play a key role in plant protection. Its conjugate naringenin-7-*O*-glucoside was pinpointed for its higher concentration in some barley genotypes resistant to FHB [20,21] such as kaempferol and kaempferol glucosides the biosynthetic pathway of which includes naringenin as precursor. Similarly to naringenin and its derivatives, several reports support the contribution of catechin to plant resistance against *F. graminearum*. Catechin concentration was shown to increase in some resistant naked barley seeds following *Fusarium* inoculation [56] and catechin was highlighted for its higher amounts in FHB resistant two-row barley genotypes compared to susceptible ones [30]. Several studies have also illustrated the potential impact flavonoids could exert on mycotoxin production. Various reports describe the ability of flavonoids to inhibit aflatoxin [57,58] or patulin production [59]. Their effect on TCTB biosynthesis has, however, been poorly documented with the exception of the publication of Desjardins *et al.* [60] that describes an inhibitory effect of flavones on the biosynthetic step that catalyzes the conversion of trichodiene (the first chemical intermediate in trichothecene biosynthesis) to oxygenated trichothecenes that contain a 12,13-epoxy group.

#### 3.1.2. Non Flavonoid Phenylpropanoids: Phenolic Acids and Derivatives

As shown in Table 3, various metabolites putatively assigned to phenolic acids, which represent the most common form of phenolic compounds in cereal kernels [61], were highlighted for their potential contribution to chemical resistance of wheat or barley against DON-producing fungi. These metabolomic data support the results of several previously published studies aiming at investigating the contribution of phenolic acids to cereal resistance that showed that resistance to GER and/or FHB may be linked with high contents of phenolic acids in mature maize [62,63,64], wheat [65,66] and barley [56] kernels. It is widely acknowledged that, in response to pathogen infection, phenolic acids are released from the cell wall or massively synthesized by the plant to accumulate rapidly at the site of infection [67]. Phenolic acids thus operate in defense response through direct interference with the fungus [68] or through the reinforcement of plant structural components to act as a mechanical barrier against the pathogen. Alternatively, their demonstrated *in vitro* ability to inhibit mycotoxin production [69] indicates that they may also specifically reduce mycotoxin accumulation *in planta*.

In kernels, phenolic acids exist as soluble free acids or as soluble conjugates that are esterified to sugars, and as insoluble bound forms. According to Fernandez-Orozco *et al.* [61], 75% of the total phenolic acids are bound to structural components of the cell wall in wheat kernels. In the published metabolomic studies that have addressed FHB resistance, no alkaline step was included in the extraction protocol of plant metabolites and only soluble forms of phenolic acids were considered. Table 3 emphasizes the potential contribution of cinnamic acid and its hydroxylated derivatives including p-coumaric, caffeic, ferulic and sinapic acids and their sugar-esterified forms in wheat and barley resistance to FHB. In addition, metabolites putatively assigned to 4-coumaroylshikimate [24] and 4-coumaroylquinate [26] in wheat and a metabolite assigned to rosmarinic acid in barley showed increased levels in some resistant genotypes inoculated with *F. graminearum*. Ferulic acid accounts for the highest level of hydroxycinnamic in grains [61]. Most of the studies related to the contribution of ferulic acid to the resistance of cereals against *F. graminearum* have focused on bound forms, cross-linked to polysaccharides by ester bonds and to components of lignin by ether bonds, and their structural role in cell wall [62,63,65]. In maize, Bily *et al.* [63] concluded that ferulic acid and its dehydromeric forms act as resistance factors to *F. graminearum* through type I resistance (resistance to initial penetration) and type II resistance (resistance to propagation due to a lower degradability of the cell wall). In addition, several studies have reported the ability of ferulic acid to inhibit *Fusarium* growth [62,64,65,66,70] and mycotoxin production [69,71,72]. The potential contribution of *p*-coumaric acid to FHB resistance was supported by metabolomic studies in barley [29] and wheat [26], in accordance with the results of targeted approaches in wheat tissues [27] and flax seedlings [73]. *p*-Coumaric acid plays a key role in plants as the starting point for multiple branching of the phenylpropanoid pathway, leading to the synthesis of lignins, lignans, anthocyanins and other phenolic acids. *p*-Coumaric acid was also shown to significantly reduce the *in vitro* biomass of *F. graminearum* [20,70]. Interestingly, Siranidou *et al.* [66] indicated a synergistic antifungal effect of *p*-coumaric with ferulic acid. Similar to ferulic and *p*-coumaric acids, caffeic acid, for which a potential role in resistance was highlighted by Kostyn *et al.* [73], is a potent inhibitor of *Fusarium* growth [63,70] and of DON production [74]. Surprisingly, sinapic acid, although reported as a predominant form of soluble conjugated phenolic acids in wheat and highlighted for its potential contribution in chemical defense against *F. graminearum* in wheat and barley (Table 3), has been poorly studied for its antifungal and “anti-mycotoxin” properties. Regardless of the phenolic compounds, the exact mechanism by which mycotoxin production is inhibited remains unclear. The results of Ponts *et al.* [70] indicated that while the lipophilic properties of phenolic acids were primary factors of their antifungal action, their antioxidant properties were tightly linked with their efficiency to modulate the production of DON. Because DON accumulation was shown to be enhanced by a peroxide stress [75], the inhibition of DON production by phenolic acids is consistent with their reported ability to scavenge reactive oxygen radicals. Moreover, according to Passone *et al.* [76], antioxidant compounds may interfere with mycotoxin production via a nonspecific mechanism, involving the perturbation of membrane function and leading to a modification of its permeability. Lastly, the results of Boutigny *et al.* [69] that indicated a down-regulation of the expression of the genes involved in DON biosynthesis by *F. graminearum* when ferulic acid was added to *in vitro* culture media are in accordance with a transcriptional control exerted by phenolic acids.

**Table 3:** Metabolites pinpointed for their potential contribution to FHB resistance and putatively assigned to non flavonoid phenylpropanoid compounds.

Subgroup	Putative Name of Identity	Reference
Phenolic acid and derivatives	1-*O*-Sinapoyl-β-d-glucose	[24]
	5-*O*-Feruloylquinic acid	[21]
	6′-*O*-(*p*-Coumaroyl)-procumbide	[25]
	7-*O*-(4-Methoxycinnamoyl) tecomoside	[21]
	7-*O*-Glucoside-ferulic acid	[21]
	Anisic acid	[32]
	Benzene acetic acid	[32]
	Benzoic acid	[32]
	Caffeic acid glycoside	[25]
	Chlorogenic acid	[21]
	Cinnamic acid	[29,32]
	Feruloyl-3-(arabinosylxylose), *cis*-*p*-coumaric acid 4-[apiosyl-(1->2)-glucoside]	[25]
	Cyano-*p*-hydroxycinnamic acid	[29]
	Diferulic acid	[25]
	Ferulic acid	[26]
	Geranyl cinnamic acid	[25]
	Methyl 6-*O*-*p*-*trans*-coumaroyl-β-d-glucopyranoside	[21]
	Methyl cinnamic acid	[30]
	*m*-Hydroxycinnamic acid	[27]
	*p*-Coumaric acid	[21,29,30]
	*p*-Coumaroylquinic acid	[26]
	*p*-Coumaroylshikimic acid	[24]
	*p*-Methoxycinnamic acid	[21]
	Rosmarinic acid	[30]
	Salvianolic acid	[31]
	Sinapic acid	[21,26]
	α-Cyano-*p*-hydroxycinnamic acid	[30]
	β-d-Glucopyranosyl-caffeic acid	[26]
	β-d-Glucopyranosyl-sinapic acid	[21]
Phenolic alcohol	1-β-(3-Hydroxy-4,5-dimethoxyphenyl)-*O*-glucopyranoside	[21]
	3,4-Dihydroxystyrene	[29]
	Caffeoyl alcohol	[30,31]
	Catechol	[29]
	Coniferin	[21,25,29]
	Coniferyl alcohol	[26]
	Dihydroconiferyl alcohol glucoside	[24]
	Dihydrodiconiferoyl alcohol	[21]
	Gingerol	[30]
	Guaiacol	[29]
	*p-*Coumaryl alcohol	[29]
	*p*-Hydroxycinnamyl alcohol 4-d-glucoside	[25]
	Pyrogallol	[21]
	Sinapoyl alcohol	[25,29]
	Syringin	[24,26]
	*trans*-*p*-Ferulyl alcohol 4-*O*-[6,[6-(2-methyl-3-hydroxypropinyl)] glucopyranoside	[21,29]
Phenolic aldehyde	Coniferaldehyde	[29]
	Caffeyl aldehyde	[29]
	Sinapaldehyde	[25,26,29]
	Sinapaldehyde glucoside	[21]
Lignan and stilbene	(+)-Pinoresinol 4-*O*-(6-*O*-galloyl)-β-d-glucopyranoside	[25]
	(+)-Pinoresinol	[25]
	(+)-Syringaresinol-*O*-β-d-glucoside	[21]
	1-Acetoxypinoresinol	[21]
	3,4′,5-Trihydroxystilbene 4′-*O*-β-d-(6′′-*O*-galloyl) glucopyranoside	[31]
	4′-Demethylpodophyllotoxine	[30]
	4′-Prenyloxyresveratrol	[21]
	5-Methoxypodophyllotoxin	[30]
	6-Methoxypodophyllotoxin	[24]
	Astringin	[21]
	Bisosthenon	[31]
	Deoxypodophyllotoxin	[21]
	Diphyllin	[30]
	Hydnocarpin	[31]
	Matairesinol	[21]
	Medioresinol 4′-*O*-β-d-glucopyranoside	[21]
	Oxyresveratrol 2-*O*-β-glucopyranoside	[30]
	Phyllanthusmin B	[21]
	Podorhizol β-d-glucoside	[21,31]
	Secoisolariciresinol di-*O*-glucoside	[21]
	Threo-carolignan E	[25]
	Tuberculatin	[31]
Coumarin	4-Geranyloxy-5-methyl coumarin	[31]
	5-Methoxyfuranocoumarine	[29]
	6,7-Dihydroxy-5-methoxycoumarin 6-β-d-glucopyranosyde	[21]
	Coumarin	[29,32]
	Dimethoxy 4-phenlycoumarin	[30]
	*trans*-Grandmarin isovalerate	[21]

#### 3.1.3. Non-Flavonoid Phenylpropanoids: Lignins and Lignans

Lignin of cereal species includes three types of monomers, *i.e.*, *p*-hydroxyphenyl, guaiacyl, and syringyl phenylpropanoid monolignols. In black and yellow barley cultivars with a high level of resistance to FHB, *F. graminearum* inoculation was shown to induce an increase in the concentrations of metabolites putatively identified as syringyl lignin precursors (sinapaldehyde and sinapyl alcohol) and guaiacyl lignin precursors (coniferaldehyde or caffeyl aldehyde) [29]. Consistently, a deposition of lignin in resistant wheat genotypes after *F. graminearum* inoculation was pointed out by Siranidou *et al.* [66] and Lionetti *et al.* [77] reported the occurrence of constitutive differences in monolignol composition of lignin between resistant and susceptible durum wheat cultivars. The resistant lines were characterized by a higher amount of syringyl-type lignin. Actually, lignin acts as plant defense by setting up mechanical barriers to the pathogen progression through cell wall changes which, as a result of polymerization of monolignol glucosides into syringyl-type lignin, lead to a reinforced cell wall that is more resistant to fungal cell wall degrading enzymes [25,66,78,79]. In addition, this increase in cell wall thickness allows limiting the diffusion of the pathogen-produced mycotoxins [66]. Lignans can also play a role as antifungal phytoalexins [78] as demonstrated for three lignans isolated from nutmeg seeds that showed an efficient antifungal activity against various pathogenic fungi [80].

### 3.2. Metabolites Derived from the Fatty Acid Pathway

Almost 40 metabolites putatively associated with fatty acid metabolic pathways have been identified for their potential contribution to cereal resistance against *F. graminearum* (Table 4). As summarized in the review of Kachroo and Kachroo [81], fatty acids and their derivatives play significant role in plant defense against pathogens. They are essential for basal immunity and gene-mediated resistance in plants, they participate to the induction of systemic acquired resistance and some of breakdown products, such as oxylipins, have a key role in plant defense signaling pathway.

**Table 4:** Metabolites pinpointed for their potential contribution to FHB resistance and classified as fatty acids and derivatives.

Putative Name of Identity	Reference
(−) Jasmonic methyl ester	[24]
(+)-7-*iso*-Jasmonoyl-l-isoleucine	[25,26]
(*E*)-Dodec-2-enedioic acid	[24,30,31]
10,16-Dihydroxy-hexadecanoic acid	[20,31]
10-Methyl-lauric acid	[30]
12-oxo-*cis*-10,15-Phytodienoic acid	[24]
13(*S*)-Hydroperoxy linolenic acid	[21,26]
13(*S*)-Hydroperoxy-9(*Z*),11(*E*),15(*Z*)-octadecatrienoic acid	[24]
18-oxo-Oleic acid	[24]
2(*Z*),4(*E*)-Decadienoic acid	[30]
2,3-Dinor-8-*iso*-prostaglandin F1α	[24]
2,3-Dinor-8-*iso*-prostaglandin F2α	[24]
2-Hydroxy palmitic acid	[31]
2-Methoxy-6(*Z*)-haxadecenoic acid	[30]
2-Nonadecenoic acid	[31]
3-Hydroxy-3-methylglutaric acid	[21]
3-Hydroxytetradecanedioic acid	[30]
3-oxo-2-(2-Pentenyl)-cyclopentaneoctanoic acid	[21,24]
5-Pyrophosphate mevalonic acid	[21]
7-Dehydrologanin tetra acetic acid	[24]
8-oxo-9,11-Octadecadiynoic acid	[21,29]
9,10-Epoxy-18-hydroxystearic acid	[24]
9-oxo-Nanoic acid	[24]
9(*S*)-Hydroxy-10(*E*),12(*Z*)-octadecadienoic acid	[26]
Acide-5,7-nonadienoique	[29]
Adipate	[29]
Decenedioic acid	[29,30]
Dihydroxylinoleic acid	[29]
Dioxo-decanoic	[28]
Dodecanedioic acid	[30]
Dodecanoic acid	[30]
Eicosanoic acid	[21,24]
Glycerol	[21]
Heptadecanoic acid	[21,29]
Heptadetrienoic acid	[31]
Heptenoic acid	[30]
Hexadecanoic acid	[24]
Jasmonic acid	[21,23,26,30,31]
Jasmonoyl-valine	[25,26]
Linoleic acid^a^	[24,30,31]
Linolenic acid	[21,24,26,30,31]
Octadeacnoic acid	[1]
Oleic acid	[21,30,31]
ω-Hydroxydodecanoic acid	[20]
Tetradecanoic acid	[30]
Tuberonic acid glucoside	[24]
Undecanoic acid	[20,21]

#### 3.2.1. Oleic, Linoleic and Linolenic Acid

The unsaturated C18:1, C18:2 and C18:3 fatty acids, oleic, linoleic and linolenic acid, have been reported as constitutive defense metabolites in barley [21,30], supporting previous studies that have illustrated their role in plant resistance against fungal pathogens such as resistance of bean against *Botrytis cinerea* [82]. These fatty acids have been shown as antimicrobial compounds able to limit the growth of fungal pathogens [83] including toxigenic ones such as *F. graminearum* [30] and *Aspergillus parasiticus* [84]. Several reports have also indicated that fatty acids were able to modulate the production of mycotoxins. Depending on the considered study, linoleic acid was reported as an activator [85] or an efficient inhibitor [84] of aflatoxin production by *Aspergillus parasiticus.* To our knowledge, no up-to-date data have been published concerning the modulation of DON production by fatty acids. In addition to their antimicrobial effect, fatty acids were also supposed to modulate ROS production [86] and to participate in resistance to fungal pathogens through their role in cuticle formation which constitutes a physical barrier to pathogen ingress. Nevertheless, the main role ascribed to fatty acids in plant defense against microbes results from the activity of some of their breakdown products and mostly of oxylipins.

#### 3.2.2. Oxidation Products of Fatty Acids

The plant oxylipin pathway is a major defense signaling pathway. The biosynthesis of oxylipins begins with the oxidation of free polyunsaturated fatty acids, chiefly linoleic (C18:2) and linolenic acid (C18:3), through the action of lipoxygenases. The main plant lipoxygenases are referred to as 9-LOXs and 13-LOXs as oxidation occurs either at the position 9 or 13 of the carbon chains, respectively. The resulting two fatty hydroperoxides induce two distinct biosynthetic pathways. 13-LOXs products lead to the formation of jasmonic acid and its derivatives. 9-LOXs products lead to less known metabolites but numerous studies on different fungal/host interaction suggest their implication as defense factors in response to fungal attack [87]. For instance, activation of the 9-LOX pathway was suggested to contribute to the activation of host defense responses against fungal pathogens in mycorrhizal plants [88]. However, in other plant/fungus pathosystems involving toxigenic fungi, 9-LOXs products were reported as factors of host susceptibility. By generating maize mutant lines in which the function of a 9-LOX gene was abolished, Gao *et al.* [87,89] observed that inactivation of this 9-LOX gene led to an increased susceptibility of maize to *Aspergillus flavus* and *Aspergillus nidulans* but also to *F. verticillioides*. When inoculated on kernels from the mutant lines, the former species were shown to produce more conidia and mycotoxins (aflatoxins for both *A. flavus* and *A. nidulans* and Fumonisin B1 for *F. verticillioides*), in accordance with previous *in vitro* results that have demonstrated an enhancing effect on aflatoxin production exhibited by 9[*S*]-hydroperoxy-*trans*-10, *cis*-12-octadecadienoic acid or 9S-HPODE [90,91,92]. According to Brodhagen and Keller [93], plant 9-LOXs products, by mimicking the fungal oxylipins called psi factors for precocious sexual inducers, could be sensed by the fungus itself to regulate mycotoxin biosynthesis and sporulation. Concerning the production of DON by *F. graminearum*, 9S-HPODE was recently ascribed as a potential toxin-conductive factor in wheat by Nobili *et al.* [94]; further investigations are however required to demonstrate this effect. Whereas 9S-HPODE was shown to promote mycotoxin production by some fungal species as detailed above, 13S-HPODE exhibited a significant inhibitor effect on aflatoxin biosynthesis [84,93]. *In* *planta*, the conversion of 13S-HPODE by the action of an allene oxide synthase leads to the biosynthesis of compounds from the jasmonate family including jasmonic acid and methyl jasmonate.

#### 3.2.3. The Signaling Molecules, Jasmonic Acid and Its Derivatives

As summarized in Table 4, several metabolomic studies have highlighted the involvement of jasmonic acid [21,23,26,30] and methyl jasmonate [24] in resistance to DON-producing *Fusarium* species. In addition, accumulation of (+)-7-*iso*-jasmonoyl-l-isoleucine formed by the conjunction of jasmonic acid and isoleucine was shown to be induced in wheat rachises and spikelets following *F. graminearum* inoculation, just like that of jasmonoyl-valine (in rachises only) [25,26]. Furthermore, the induction of the jasmonic acid signaling pathway in *F. graminearum* inoculated wheat was demonstrated by the transcriptional studies performed by Li and Yen [95] who reported an up-regulated expression of several jasmonic-acid responsive genes including an allene oxidase. In barley, according to Kumaraswamy [29], when the activation of jasmonate signaling was observed following inoculation with a DON-producing *F. graminearum* strain, this was not the case with a non-producing one, which could indicate that the DON and not the fungal infection is responsible for this plant defense reaction. The ability of DON to induce jasmonate signaling was also discussed by Gunnaiah *et al.* [26] who suggested that by inducing jasmonate signaling, DON activates the production of hydroxycinnamic acid amides that participate to cell thickening.

Jasmonic acid and methyl jasmonate are well known for their roles as plant stress hormones, causing programmed cell death activation and other *in vivo* defense mechanisms as the production of reactive oxygen species [ROS] or the deposits of wax layers on plant tissues [96]. Moreover, jasmonic acid and its derivate methyl jasmonate positively regulate the phenylpropanoids pathway [97] that is a central pathway in resistance to *F. graminearum* as detailed above. Resistance against necrotrophic pathogens was shown to generally require the activation of the jasmonate signaling pathway. This was demonstrated for the plant necrotrophic pathogen *Botrytis cinerea* that colonizes senescent or dead plant tissues of a broad range of hosts including tomato, potato or grape [98,99]. Although *F. graminearum* is a necrotrophic plant pathogen, a biotrophic phase has been described at early stages of infection during which the fungus does not penetrate host cells and is contained in extracellular spaces [1]. How wheat can utilize jasmonate signaling pathway to control *F. graminearum* infection was recently clarified by Makandar *et al*. [100]. The previous authors demonstrated that jasmonate signaling pathway has two contrasting roles in the interaction of wheat with *F. graminearum*: jasmonic acid promotes disease by constraining the salicylic acid signaling pathway during the early stage of infection and promotes resistance during the later stages of infection. Moreover, external application of jasmonic acid has been shown to activate glucosyltransferase in *Arabidopsis thaliana* [101] and barley [30], which is a key enzyme activity involved in a DON detoxification pathway that transforms DON into less phytotoxic DON-3-*O*-glucoside.

In addition to their major role in plant defense signaling pathway, jasmonic acid and methyl jasmonate were shown to exhibit antimicrobial properties towards toxigenic fungi such as *A. flavus* [102] and *F. graminearum* [30]. Methyl-jasmonate was also reported as able to modulate aflatoxin production in a dose-dependent manner [103], resulting in an inhibition [84] or an enhancement [104] effect. To our knowledge, no previous data describing the direct impact of jasmonic acid and derivatives on DON-producing fungi have been published until now.

### 3.3. Terpenoids

Terpenoids (or isoprenoids) are considered as the largest and most diverse class of plant secondary metabolites. They are produced in plant cells via the mevalonate and methylerythritol 4-phosphate pathways. Plant terpenoids include compounds ranging from C5 hemisesquiternes to C40 tetraterpenes, with diverse physical and chemical properties leading to lipophilic or hydrophilic, volatile or non-volatiles metabolites. Thus, depending on the chosen extraction protocol and the analytical equipment, many of them may have not been considered in metabolomic analyses of plant tissues. Compilation of wheat and barley metabolomic studies aiming at identifying the biochemical defense compounds involved in resistance to *F. graminearum* or DON results in a list of 30 compounds putatively identified as terpenoids, equally distributed among constitutive and induced metabolites (Table 5). Abscisic acid and five related compounds were classified as induced metabolites. The reports of Kumaraswamy *et al.* [29] and Gunnaiaha *et al.* [26] indicated that *F. graminearum* inoculation induced an increased accumulation of abscisic acid in wheat and barley, in accordance with the study of Petti *et al.* [105] that showed a production of abscisic acid in barley head tissues inoculated with *F. culmorum* as early as 4 h post-pathogen treatment. There is growing evidence for an active and important role of abscisic acid signalling in plant-pathogen interactions [106]. Regarding FHB resistance, the role of abscisic acid can be linked with its regulatory effect in callose deposition [107]. Indeed, deposition of cell wall polymer callose in the transition zone of the spikelet’s rachilla and rachis was shown to be one component of type II resistance in wheat and barley, *i.e.*, resistance against the spread of the pathogen within the plant-host [108]. Besides, the involvement of abscisic acid in FHB resistance can also be linked to its negative interaction with the signaling ethylene pathway [107] since, according to Chen *et al.* [109], *F. graminearum* exploits ethylene signaling to enhance colonization in wheat tissues. Lastly, the possibility that abscisic acid could limit *F. graminearum* penetration through its control of stomatal aperture cannot be omitted [106]. In addition to abscisic acid derivatives, several iridoids such as asperuloside [24] and its precursor loganine [21,26], were pointed out as metabolites potentially involved in wheat and barley FHB resistance. This was also the case for the diterpene phytocassane in barley [21] and hallalactone in wheat. These two last groups of diterpenoids have been well characterized as phytoalexins in rice and maize [110] with an antifungal activity linked to their ability to interfere and disrupt membranes. Volatile organic compounds such as linalool derivatives (Table 4) were also shown to be released by barley [21] in response to F. graminearum infection, supporting previously published data on wheat, oat and barley [111]. The mechanisms by which this induction of volatile organic compounds, known to be regulated by the jasmonic acid signaling pathway [112], contribute to plant resistance has yet to be documented. It is assumed that, similarly to other terpenoids, volatile organic compounds could also act as antifungal metabolites.

**Table 5:** Metabolites pinpointed for their potential contribution to FHB resistance and putatively assigned to terpenoid compounds.

Subgroup	Putative Name of Identity	Reference
Abscisic acid derivatives and precursors	8′-Hydroxyabscisate	[26]
	Abscisic alcohol	[26]
	Abscisic aldehyde	[26]
	Abscisic acid glucose ester	[26]
	Xanthoxin	[26,29]
Iridoids and derivatives	10-Hydroxyloganin	[26]
	7-Deoxyloganic acid	[26]
	Asperuloside	[26]
	Aucubin	[21,26]
	Deutzioside	[26]
	Ipolamiide	[24]
	Iridotrial glucoside	[24]
	Loganin	[26]
	Secologanin	[24]
Hemiterpene	Isovaleroyloxylinalool	[21]
Diterpene	Phytocassane B	[21]
	16-Diacetoxy-7a-hydroxy-18-malonyloxyent-cleroda-3-ene	[21]
	Briaexcavatin O	[21]
	Brusatol	[29]
	Hallactone B	[24]
	Inumakilactone A glucoside	[24]
	Isobrucein A	[24]
	*trans*-Communic acid	[24]
Triterpene	Cineracipadesin F	[24]
	Quinovic acid	[21]
	Salannin	[29]
Sesquiterpene	2-oxo-6-Dehydroxyneoanisatin	[24]
	3-Hydroxy-15-dihydrolubimin	[24]
	Cryptomeridiol	[21]
	Eupacunolin	[24]
Tetraterpene	Apo-13-zeaxanthione	[30]

### 3.4. Amino Acids and Derivatives

Metabolomic studies related to FHB resistance clearly indicate that increased levels of several amino acids can be correlated with increased disease tolerance. Some of these amino acids showed higher levels in resistant lines compared to susceptible ones or elevated concentrations as a response to toxigenic *Fusarium* and/or DON treatment. This was the case for phenylalanine, the concentration of which was found to be more elevated in resistant barley genotypes compared to susceptible ones and was also shown to increase following *Fusarium* treatment [29]. Similarly to phenylalanine, glutamine, aniline, proline and glycine showed higher amounts following pathogen inoculation in wheat and barley [23,27,30]. In addition, threonine, asparagine and ornithine were described as constitutive defense metabolites in wheat [32]. Lastly, a metabolite putatively identified as a glycine methyl ester clearly showed a higher concentration following wheat inoculation with *F. graminearum* with an abundance fold change ratio of 29 [21]. The potential contribution of amino acids in induced chemical defense reflects the previous work of Zhou [113] who demonstrated that several proteins involved in amino acid biosynthesis were up-regulated following *F. graminearum* inoculation in wheat. However, in wheat leaf tissues, Brown and Brindle [22] reached opposite conclusions, reporting that increased correlations of glutamine and alanine, which are a main source of nitrogen for fungi, correlated with increased disease susceptibility.

The involvement of aromatic amino acids such as phenylalanine, tyrosine and tryptophane in resistance to DON-producing *Fusarium* can be directly related with their role as precursors for a wide range of secondary metabolites that play a key role in plant defense against biotic stresses [114]. Thus, phenylalanine serves as a precursor of the phenylpropanoid pathway that begins with the conversion of phenylalanine to *trans*-cinnamic acid. The tyrosine can be catabolized into a variety of secondary metabolites including tocopherols and tocotrienols, and tyramine that is the precursor of cell wall-bound hydroxycinnamic acid amides. In addition tyrosine was also suggested to participate to the biosynthesis of some phenylpropanoids [114]. Lastly, catabolism of tryptophan leads to many indole-containing secondary metabolites such as indole-3-acetic acid (auxin), glucosinolates and terpenoids, *i.e.*, three classes of compounds largely documented for their implication in plant-pathogen interactions including wheat or barley-*F. graminearum* or *F. culmorum* interactions [105]. Similarly, the role of isoleucine in FHB resistance can directly result from its combination with jasmonic acid leading to jasmonoyl-isoleucine that plays a key function in jasmonic acid–mediated signal transduction [115]. Since threonine and methionine serve as substrate for isoleucine biosynthesis, their highlighted potential implication in resistance against toxigenic *Fusaria* is consistent with that of isoleucine. An additional role of methionine results from its conversion into *S*-adenosyl-methionin that plays a central and crucial role in several plant processes such as DNA replication and methylation and the biosynthesis of ethylene and of a variety of plant defense secondary metabolites. Threonine can also participate to plant-pathogen interactions through its phosphorylation and dephosphorylation, a key element of the regulation of plant signal transduction pathway participating to the recognition of pathogens [116].

### 3.5. Amine and Polyamine Pathway Metabolites

Previous studies have indicated that polyamines can play a key role in the response of plants to pathogens [117]. In plant cells, polyamines can occur either in free forms or conjugated to phenolic acids, mainly to ferulic, *p*-coumaric and caffeic acid (hydroxycinnamic acid amides) or bound with macromolecules including proteins and components of the cell wall. Metabolomic studies related with FHB resistance have also highlighted the potential implication of a set of metabolites putatively assigned to polyamines in wheat or barley response to DON-producing *Fusarium* strains. Free forms such as putrescine and spermine were reported to be more abundant in the resistant Sumai3 wheat genotype than in the susceptible Roblin one [32] whereas several hydroxycinnamic acid amides (*p*-coumaroyl putrescine, feruloylputrescine, *cis-p*-coumaroylagmatine, cinnamoylserotonin, feruloylagmatine, *p*-coumaroylserotonine and feruloylserotonin) showed an elevated level in wheat rachises of resistant cultivars following *Fusarium* inoculation. This was also the case for a metabolite assigned to caffoeylserotonin, an increased concentration of which was induced in wheat spikelets by *Fusarium* treatment [26]. Previous study on maize has also pointed out an implication of a variety of polyamines in response to *F. graminearum*, such as cadaverin [118]. Moreover the recent study of Wojtasik *et al.* [119] reports an increased levels of expression for a variety of genes involved in polyamine biosynthesis after flax infection by *F. graminearum*, leading to a significant increase in polyamine level in plant tissues. Currently, despite numerous studies to profile variations in polyamine levels between resistant and susceptible cultivars in response to pathogens [120] that also indicated that changes in polyamine metabolism represent a key adaptive response of plants to biotic stresses, the precise mechanisms underlying the role of polyamines in the resistance of plants to fungal pathogens remain incompletely understood. One of the most commonly accepted hypotheses is based on the ability of polyamines (free and hydroxycinnamic acid amides) to bind to cell wall components, resulting in a strengthening of the physical barrier that prevents or reduces fungal infection. Increasing evidences also suggest that through their oxidation and the generation of H_2_O_2_, polyamines, and mainly spermine, can act as mediators of plant defense activation [117]. Besides, a few studies indicate the occurrence of relationships between polyamines and plant defense hormones during plant biotic stress and that polyamines may interfere with ethylene, salicylic acid and abscisic acid metabolisms and *vice-versa* [120]. There are also a few investigations that have addressed antifungal activities of free polyamines and hydoxycinnamic acid amines [117]. The recent report of Wojtasik *et al.* [119] clearly demonstrated the ability of putrescine, spermine and spermidine to restrict the *in vitro* growth of *F. culmorum*, using concentrations of polyamines that, however, largely exceed the physiological ones. In addition, cinnamoylagmatines are direct precursors of hordatines, which have long been known to be antifungal compounds accumulating in young barley seedlings [121]. Lastly, it should not be overlooked that polyamines are also essential metabolites and a source of nutrients for invading pathogens, involved in a variety of fungal cell functions, from growth to development and differentiation. Therefore, despite the above considerations, the relationship between polyamine contents and plant resistance is not so clear, and contradictory information describing a negative role played by polyamines in plant resistance has been published leading to the proposition of strategies based on the use of polyamine biosynthesis inhibitors as a mean of control of fungal pathogens. Moreover, several reports have indicated that some microorganisms are able to perturb plant polyamine metabolism in order to adjust it to their own requirements. This could be the case for *F. graminearum* when infecting wheat. Indeed, it has been hypothesized that *F. graminearum* senses polyamines as a signal to trigger the production of DON and that intermediates of the polyamine pathway enhance the accumulation of the toxin [122]. Accordingly, a recently published report describes the use of polyamine biosynthesis inhibitors as a promising approach for the control of *F. graminearum* and DON contamination of wheat [123].

### 3.6. Carbohydrate Pathway Metabolites

Metabolic profiling of wheat spikelets revealed higher abundance of a few metabolites putatively identified as sugars that could account for wheat resistance to *F. graminearum* and DON accumulation. Concentrations of fructose have been reported to be higher in some resistant genotypes than in susceptible ones [27]. Moreover, levels of mannose, galactopyranose and myo-inositol were shown to significantly increase in a few set of resistant wheat cultivars challenged with *F. graminearum* [27,32]. Accordingly, but addressing another pathosystem, Campos-Bermudez *et al.* [118] have reported higher levels of glucose, fructose, galactose and sucrose in maize inbreds resistant to *F. verticillioides* compared to susceptible ones. The potential contribution of *myo*-inositol to wheat resistance against FHB is consistent with its function as a building block of a variety of *myo*-inositol containing molecules that play key roles in signal transduction. The most significant discoveries on the inositol signaling pathway were recently reviewed by Gillapsy [124]. In the context of plant-pathogen interactions, there is increasing evidence that inositol signaling can regulate plant hormone receptors, as well as participate to fungal recognition and to the mediation of plant wound responses. Moreover, plant cell walls contain high molecular mass polysaccharides linked in a network of ionic and covalent bonds that provide a physical barrier for pathogen ingression. The oxidation product of *myo-*inositol, d-glucuronic acid that was pinpointed by Paranidharan *et al.* [32], participates to the biosynthesis of cell wall pectic noncellulosic compounds [125] and, in some organisms, serves as a precursor for ascorbic acid [126]. Thus, *myo*-inositol synthesis and catabolism impact metabolites involved in many different and critical plant biochemical pathways involving pathways that can contribute to resistance of wheat against *F. graminearum*.

## 4. Conclusions

Cereal diseases caused by pathogenic and toxigenic *Fusarium* species are responsible for major economic damage every year. The developments of strategies to avoid *Fusarium* and mycotoxin contamination require a thorough understanding of the interactions between the cereal plant and the fungal pathogen. Despite being a relatively new approach in plant biology, metabolomic is reported as a promising and powerful tool to unravel plant-pathogen interactions. Indeed, whilst studying gene and protein expression allows anticipating the capacity of plant to respond to a biotic stress, addressing the metabolome allows investigating the “true” response of the plant taking into account gene and protein expression and the impact of environmental conditions. This review has covered the major classes of metabolites that recent metabolomics studies have pointed out for their potential contribution in resistance of wheat and barley against *F. graminearum* infection and DON accumulation. We clearly illustrated that resistant cultivars of cereal can implement a wide range of different biochemical responses participating to a complex and integrated network of events with the aim to counteract *F. graminearum* infection and spread.

**Figure 2:**
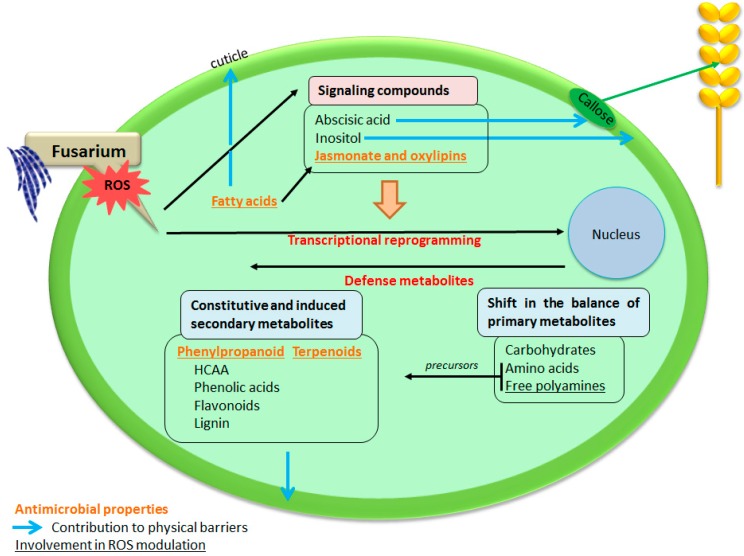
Overview of the key metabolites cereals could employ to counteract *F. graminearum*. (ROS: Reactive Oxygen Species, HCAA: Hydroxycinnamic Acid Amides). Compounds in bold orange are compounds with antimicrobial properties, underlined are contributing to ROS modulation and the blue arrows symbolize the participation to reinforcement of physical barriers.

As summarized on Figure 2, this network starts with the perception of the invading fungal pathogen and ends with the accumulation of soluble antifungal compounds and wall-bound, barrier-forming structures. A large set of signaling compounds and plant hormones play a pivotal role; activated as soon as the pathogen is recognized, they orchestrate the transcriptional reprogramming of the infected plant cell. Figure 2 also clearly indicates that when the accumulation of various secondary metabolites is one of the major responses of the plant cell, carbohydrates and amino acid metabolism also significantly participate to this arsenal of chemical defenses.

However, metabolomic approaches are facing major difficulties. One of the main critical limitations directly results from the great amount of generated data and the current inability to properly annotate many of the detected plant metabolites despite the accessibility to public databases including HMDB, NIST, KEGG, MassBank and METLIN [21,30,32,127]. Whilst the previous databases represent a useful tool to annotate primary metabolites, they are currently not comprehensive enough for a relevant annotation of plant secondary metabolites, which still represents a challenging task. Therefore, a large part of metabolites identified in current metabolomic studies remains unidentified. Moreover, profiling all plant metabolites simultaneously is extremely challenging due to the high chemical diversity and complexity of the plant metabolome. Currently no extraction protocol combined with a single analytical technique allows considering the entire metabolome and consequently, the data delivered by metabolomic studies only cover a fraction of the metabolome. This barrier can be partially overcome through the use of combined selective extraction protocols and a set of complementary analytical technologies. Indeed, the combination of NMR and MS techniques was recently demonstrated to be a powerful strategy for a comprehensive analysis of the metabolome of urines from mice [127]. Besides, when considering mechanisms of plant resistance against biotic stresses, it should not be overlooked that in addition to chemical defenses, physical and morphological ones are also involved. Thus, whilst metabolomic studies can be highly relevant to address the chemical traits of cereal defenses against *F. graminearum* and DON accumulation, they do not consider the two additional components. Large sets of polymers involved in the reinforcement of cell walls that require being fragmented in oligomers and monomers before analysis totally escape to the metabolomic analytical strategy [128].

Nevertheless, metabolomic analysis show promising opportunities for plant breeding through the identification of metabolic markers, which use, in combination with genetic markers, can lead to extremely powerful selection tools. Compared to molecular markers, biochemical ones have the advantage to be more closely linked to the phenotype. However, whilst the genetic background is stable under any environment, metabolic profiles are strongly impacted by environmental and experimental conditions, resulting in significant constraints that require to be carefully considered for the validation of metabolic markers. With regards to the selection of cereal cultivars resistant to *F. graminearum* and less sensitive to mycotoxin contamination, the metabolomic studies gathered in this review (summarized in Table 1) clearly illustrate the promising interest this approach could provide. Today the breeding industry continues to face significant problems in the selection of resistant cereal lines to *Fusarium* diseases. Because FER and GER resistance are polygenic traits, genetic studies encountered major limitations. In the cases of such complex traits, the predictive power of genetic data can be significantly improved by combining it with metabolic measurements, as previously highlighted by Gartner *et al.* [129]. As of now, several putative metabolite markers of FHB and GER resistance, which mainly belong to the phenylpropanoid pathway, have been highlighted. These putative markers gather essential criteria including: (i) a putative identification according to public databases; (ii) a significant high change in abundance in the FHB-resistant genotype compared to the susceptible one; (iii) a validation taking into account different FHB-resistant genotypes; (iv) a biological meaning according to known plant defense mechanisms [29]. However, before being exploited in plant breeding strategies, these potential biomarkers require validation. This validation can be achieved through the mapping of the potential markers in metabolomic pathways and the identification of catalytic activities and genes involved in their biosynthetic pathway [128]. Silencing or overexpression of the so identified genes will allow demonstrating their role in resistance against *F. graminearum*. However, since the levels of plant metabolites usually depend on a number of genes, reverse genetic approaches might be challenging for the validation of certain metabolites. An additional strategy could rely on genetic and QTL colocation approaches. Investigating the position of metabolites QTL and comparing these locations with that of FHB resistance QTL can provide further arguments in favor of the role played by a metabolic pathway in cereal resistance to *F. graminearum*. Actually, the mechanisms of resistance governed by the FHB resistance locus, Fhb1, was recently investigated by Gunnaiaha *et al.* [26] and Kluger *et al.* [18] who demonstrated that this locus was mainly associated with cell wall thickening and DON detoxification pathways.
